# Genetic Testing Resources and Practice Patterns Among Pediatric Cardiomyopathy Programs

**DOI:** 10.1007/s00246-024-03498-6

**Published:** 2024-05-07

**Authors:** Justin Godown, Emily H. Kim, Melanie D. Everitt, Wendy K. Chung, Irene D. Lytrivi, Sonya Kirmani, Paul F. Kantor, Stephanie M. Ware, Jean A. Ballweg, Ashwin K. Lal, Neha Bansal, Jeffrey Towbin, Steven E. Lipshultz, Teresa M. Lee

**Affiliations:** 1 Division of Pediatric Cardiology, Monroe Carell Jr. Children’s Hospital at Vanderbilt, Nashville, TN USA; 2 BioMarin Pharmaceutical Inc, Novato, CA USA; 3 Department of Pediatrics, University of Colorado, Children’s Hospital Colorado, Aurora, CO USA; 4 Department of Pediatrics, Boston Children’s Hospital, Harvard Medical School, Boston, MA USA; 5 Department of Pediatrics, Columbia University Irving Medical Center, New York, NY USA; 6 Department of Pediatrics, University of Wisconsin School of Medicine, Madison, WI USA; 7 Department of Pediatrics, Keck School of Medicine of USC, Children’s Hospital Los Angeles, Los Angeles, CA USA; 8 Department of Pediatrics and Medical and Molecular Genetics, Indiana University School of Medicine, Indianapolis, IN USA; 9 Department of Pediatrics, Helen DeVos Children’s Hospital, Grand Rapids, MI USA; 10 Division of Pediatric Cardiology, University of Utah, Primary Children’s Hospital, Salt Lake City, Utah, USA; 11 Division of Pediatric Cardiology, Mount Sinai Kravis Children’s Hospital, New York, NY USA; 12 Heart Institute, Le Bonheur Children’s Hospital, Memphis, TN USA; 13 Department of Pediatrics, Jacobs School of Medicine and Biomedical Sciences, Clinical and Translational Research Center, University at Buffalo, 875 Ellicott Street, Suite 5018, Buffalo, NY 14203 USA

**Keywords:** Cardiomyopathy, Genetic testing, Genetic counseling, Pediatrics

## Abstract

The use of genetic testing has enhanced the diagnostic accuracy of heritable genetic cardiomyopathies. However, it remains unclear how genetic information is interpreted and incorporated into clinical practice for children with cardiomyopathy. The primary aim of this study was to understand how clinical practice differs regarding sequence variant classifications amongst pediatric cardiologists who treat children with cardiomyopathy. A secondary aim was to understand the availability of genetic testing and counseling resources across participating pediatric cardiomyopathy programs. An electronic survey was distributed to pediatric heart failure, cardiomyopathy, or heart transplantation physicians between August and September 2022. A total of 106 individual providers from 68 unique centers responded to the survey. Resources for genetic testing and genetic counseling vary among large pediatric cardiomyopathy programs. A minority of centers reported having a geneticist (*N* = 16, 23.5%) or a genetic counselor (*N* = 21, 31%) on faculty within the division of pediatric cardiology. A total of 9 centers reported having both (13%). Few centers (*N* = 13, 19%) have a formal process in place to re-engage patients who were previously discharged from cardiology follow-up if variant reclassification would alter clinical management. Clinical practice patterns were uniform in response to pathogenic or likely pathogenic variants but were more variable for variants of uncertain significance. Efforts to better incorporate genetic expertise and resources into the clinical practice of pediatric cardiomyopathy may help to standardize the interpretation of genetic information and better inform clinical decision-making surrounding heritable cardiomyopathies.

**Supplementary Information:**

The online version contains supplementary material available at 10.1007/s00246-024-03498-6.

## Introduction

The use of genetic testing has expanded rapidly in the management of pediatric cardiomyopathies. Improved understanding of the genetic basis of disease has real and potential benefits for family screening, disease prognostication, and identification of novel therapeutics which may more precisely target the underlying disease mechanism. However, heterogeneity of presentation and uncertainties surrounding the interpretation of genetic variants have significant implications for clinical practice.

The American college of medical genetics and genomics (ACMG) in conjunction with the Association for Molecular Pathology have published guidelines for the interpretation of sequence variants, with classification based upon evidence of pathogenicity including population data, computational data, functional data, and segregation data [1]. This framework provides standards for variant classification into five groups; 1. pathogenic, 2. likely pathogenic, 3. variant of uncertain significance (VUS), 4. likely benign, and 5. benign. This classification of sequence variants should directly impact clinical practice, specifically regarding cascade screening of at-risk family members. However, as additional data become available, these classifications may change, with potential clinical implications [2]. It is unknown how variant classifications are applied in the clinical practice by cardiologists treating children with cardiomyopathy. This study aimed to assess the genetic testing and genetic counselling resources across large pediatric cardiomyopathy practices and to understand how changes in variant interpretations impact clinical practice.

## Methods

This study was developed in conjunction with the pediatric cardiomyopathy registry (PCMR) study group [3–5]. An electronic survey was developed within the research electronic data capture (REDCap) environment [6]. The survey is provided in Online Resource 1.

The survey was distributed to pediatric cardiology providers via three overlapping networks between August 1st, 2022 and September 5th, 2022; 1. The Pediatric Heart Transplant Study (https://pediatrichearttransplantsociety.org/), 2. The Advanced Cardiac Therapies Improving Outcomes Network (https://www.actionlearningnetwork.org/), and 3. PediHeartNet (http://pediheart.net/). The invitation asked providers to self-identify as pediatric heart failure, cardiomyopathy, or heart transplantation providers. Multiple respondents from a single center were allowed.

Standard summary statistics were calculated. Categorical data are presented as frequency and percentage. Data are presented at the respondent-level for questions pertaining to clinical practice patterns and at the center level for questions pertaining to resource availability. For center level data when there were multiple respondents from a single institution, the most senior respondent was utilized to account for rare discrepancies between respondents from the same institution.

This study was approved by the Vanderbilt University Medical Center Institutional Review Board as well as the PCMR study group.

## Results

A total of 106 individual medical providers responded to the survey. Given the use of large networks for survey distribution with an unknown number of qualified providers invited, the response rate was unable to be calculated. Respondents were from 68 unique centers and predominantly located in the United States (*N* = 93, 88%). Other countries represented include Canada (*N* = 3), Australia (*N* = 1), Austria (*N* = 1), Italy (*N* = 1), Saudi Arabia (*N* = 1), and Spain (*N* = 1).

Center-level resource data are presented in Table 1, and respondent-level clinical practice data are shown in Table 2.

**Table 1:** Survey responses at the level of individual centers

	(*N* = 68)
Genetic testing lab	
In-house (academic medical center)	3 (4.5%)
Commercial	31 (46.3%)
Combination	33 (49.2%)
Geneticist on faculty in the division of cardiology	16 (23.5%)
Genetic counsellor within the division of cardiology	21 (30.9%)
Have a process to follow-up with patients who were previously discharged from follow-up if a variant is reclassified that would change clinical management	13 (19.0%)

**Table 2:** Survey responses at the level of individual respondents

	(*N* = 106)
Years of experience	
< 1 year	4 (3.8%)
1 to 4 years	24 (22.9%)
5 to 9 years	26 (24.8%)
10 to 14 years	27 (25.7%)
15 to 19 years	10 (9.5%)
20 or more years	14 (13.3%)
How often do you perform whole-exome sequencing on a child with cardiomyopathy	
Always	7 (6.7%)
Frequently	28 (26.7%)
Sometimes	40 (38.1%)
Not often	27 (25.7%)
Never	3 (2.9%)
Specific findings prompt consideration of whole-exome sequencing	70 (68.6%)
How often do you find that cost is prohibitive to obtaining genetic testing	
Almost Always	1 (1%)
Often	21 (20%)
Neutral	16 (15.2%)
Not often	47 (44.8%)
Almost never	20 (19%)
How reliable are genetic testing interpretations	
They are always accurate	0 (0%)
They are mostly accurate, but can rarely change	20 (19%)
They are mostly accurate, but can sometimes change	76 (72.4%)
They are mostly accurate, but can often change	9 (8.6%)
They are never accurate	0 (0%)

**Fig. 1:**
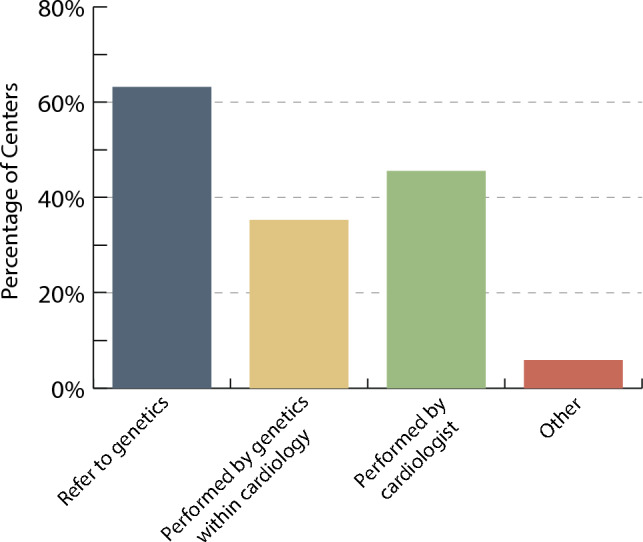
Process to access genetic testing at the center level. Respondents could select more than one process and therefore the total percentage is > 100%

**Fig. 2:**
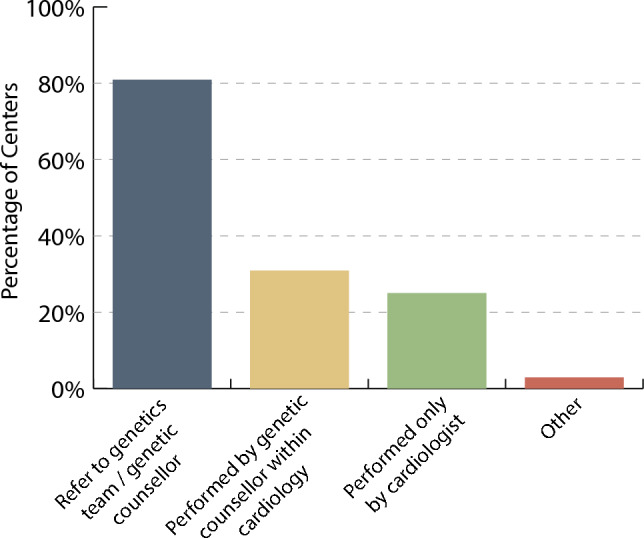
Process to access genetic counselling at the center level. Respondents could select more than one process and therefore the total percentage is > 100%

Three centers (4.5%) predominantly use in-house genetic testing, 33 centers (49.2%) use a combination of in-house and commercially available genetic testing, and 31 centers (46.3%) only use commercially available genetic testing. A total of 16 centers (23.5%) reported having a geneticist on faculty within the division of pediatric cardiology and 21 (30.9%) reported a genetic counsellor within the division. Only 9 centers (13.2%) reported having both a geneticist and a genetic counsellor within the division of pediatric cardiology. Referral to a clinical genetics service (outside the division of pediatric cardiology) was the most common method used to access genetic testing resources, but it was also common for genetic testing to be performed by the treating cardiologist (Fig. 1). Genetic counselling was most-commonly accessed through referral to the clinical genetics service or a genetic counsellor outside the division of pediatric cardiology and less commonly performed by the treating pediatric cardiologist or a genetic counsellor within the division (Fig. 2). Respondents also reported occasional use of genetic counselling services offered through commercially available genetic testing laboratories. Few centers (*N* = 13, 19%) have a formal process in place to re-engage patients who were previously cleared from pediatric cardiology follow-up if a variant were to be reinterpreted which changes clinical recommendations.

**Fig. 3:**
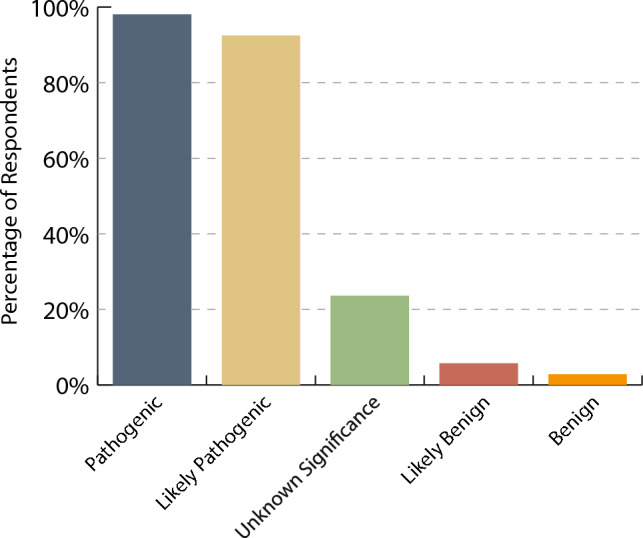
Percentage of respondents who would offer family cascade testing depending on proband variant classification

Most respondents (> 95%) would offer family cascade screening for a pathogenic or likely pathogenic variant. However, only 25 respondents (23.6%) would offer the same screening in the presence of a VUS and even fewer with benign or likely benign variants (Fig. 3).

**Fig. 4:**
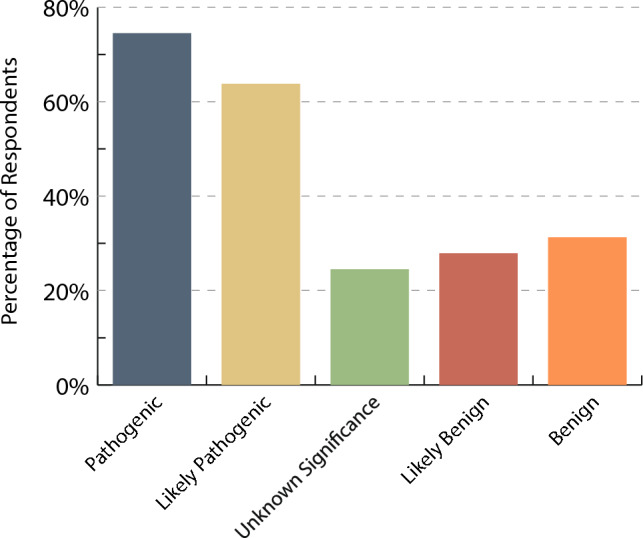
Percentage of respondents who would discharge an asymptomatic and phenotype negative relative of the proband from further cardiology follow-up if they tested negative for a familial variant depending on classification

A total of 79 respondents (74.5%) would clear an asymptomatic patient without phenotypic evidence of cardiomyopathy from further cardiology follow-up if they tested negative for a known familial pathogenic variant while 67 (63.8%) would do so with a likely pathogenic variant (Fig. 4). However, fewer providers would discharge patients from further cardiology follow-up if they tested negative for a familial VUS, likely benign, or benign variant.

There was wide variability in the use of whole-exome sequencing (WES) among respondents. Common themes emerged and there were specific clinical situations that prompted consideration of WES amongst respondents (Table 3).

**Table 3:** Respondents recommended considering whole-exome sequencing in the following situations

Strong family history
Negative cardiomyopathy genetic panel
Concern for genetic syndrome
Dysmorphic features
Multi-organ involvement/Extracardiac manifestations
Neonates with cardiomyopathy
Suspicion of metabolic or mitochondrial disease
Need for expedited results
Unusual presentation
To facilitate transplant evaluation
Developmental delays
Concern for neuromuscular disease

## Discussion

This study provides novel insights into genetic testing and genetic counselling resources among large pediatric cardiomyopathy programs and highlights variability in clinical practice in surrounding familial variant classifications. Few programs had developed resources to handle variant reinterpretations. Given the significant expansion of genetics in the clinical practice of pediatric cardiomyopathy [7], additional research is needed to optimize the implementation of genetic data in clinical practice while accounting for varying resource availability across centers.

The management of heritable cardiomyopathy requires a multidisciplinary team, of which geneticists and genetic counsellors play an integral part [8, 9]. Methods to access genetic counselling and genetic testing are variable across pediatric cardiomyopathy programs. These differences are likely secondary to varying resources across programs as well as differences in health systems and processes. Several centers reported that cardiologists assume responsibility for genetic testing as well as genetic counselling. While cardiomyopathy physicians are typically well-versed in the genetics of heritable cardiomyopathies, physicians may not have sufficient time to devote to many important aspects including identification and testing of at-risk family members, family communication, education, discussing the implications for insurance, and addressing the psychological impact of a genetic diagnosis [8]. Therefore, incorporation of genetic counsellors provides valuable expertise and represents an important component of care in the management of pediatric cardiomyopathy. Furthermore, pediatric patients with higher likelihood of neuromuscular, metabolic, or syndromic causes of cardiomyopathy, such as infants or patients with extracardiac phenotypic findings, benefit from evaluation by geneticists. This is particularly important in cases where diagnosis-based therapeutics are time sensitive [9]. Finally, only a minority of programs have systems in place to address changes in variant interpretation that may occur. Given the potential implications on patient care and risk of sudden death in some patients with cardiomyopathy, developing tools and resources to address evolving genetic information can be important clinically for the family and a source of medicolegal liability. Data are lacking to support the notion that resources within a division of cardiology are superior to external referrals; however, this may help to improve access. As centers continue to build specialized cardiomyopathy programs, integration of genetic testing and counselling within the division of pediatric cardiology represents an important consideration to improve communication and optimize the utilization of genetic information in this population.

The results of this analysis also demonstrate variability in how familial testing is used in clinical practice. Most respondents would offer cascade familial testing for a pathogenic or likely pathogenic variant in a proband. However, for a benign, likely benign, or VUS, practice is more variable. Guidelines from the ACMG recommend that VUS should not be used in clinical decision-making [1]. Lack of geneticist or genetic counselor involvement in the care of children with cardiomyopathy may contribute to the variability in practice surrounding non-pathogenic variants and the failure to align with current guidance. Whether or how centers are using these non-pathogenic variant classifications in clinical practice to determine need for serial phenotype screening of relatives remains unclear and represents an important area for improvement. This further supports the importance of having a geneticist and/or a genetic counsellor involvement in the care of children with cardiomyopathy.

Whole exome sequencing is increasingly being used to identify the genetic basis of disease [10]. While the use of whole-exome sequencing varied among respondents, there were specific clinical situations that prompted consideration of genomic testing. Rapid whole-exome sequencing can be performed in 6–15 days which may facilitate a timely diagnosis and help guide patient management for acutely ill inpatients [11]. This may also help to obviate the need for sequential testing which can be time consuming. If used as a first-or second-tier testing modality, whole-exome sequencing improves diagnostic yield at a lower cost compared to standard testing for some indications [11–13]. Understanding the pathogenicity of variants and establishing processes to effectively translate these results into clinical practice is an important consideration to optimize patient care moving forward.

There are inherent limitations to our study. Respondents self-identified as pediatric cardiomyopathy, heart failure, or heart transplant providers. While it is possible that providers with differing clinical expertise responded to our survey, there would be little incentive to do so. Given the use of large networks for survey distribution with an unknown number of qualified providers invited, the response rate was unable to be calculated. Additionally, the spectrum of clinical practice surrounding sequence variants is complex, and our survey cannot account for all potential scenarios. Centers vary in size, potentially influencing practice patterns surrounding genetic testing and/or counselling. However, questions pertaining to patient volume were not included in the survey and are not readily available to assess the impact of center size on practice. Lastly, survey respondents were predominantly from the United States. While international sites were represented, these results may not be generalizable globally given limited participation from non-U.S. centers.

## Conclusion

Resources for genetic testing and genetic counselling vary among large pediatric cardiomyopathy programs with a minority of programs having a geneticist and/or a genetic counsellor within the division of pediatric cardiology. Practice patterns are uniform for pathogenic or likely pathogenic variants but are more variable for VUS where some providers leverage these results in clinical decision-making, diverging from current ACMG guidance. Few centers have systems in place to respond to changes in variant classification that affect clinical care. Families with inherited forms of cardiomyopathy require long-term genetic follow-up to address evolving knowledge surrounding variant classification and identify at-risk individuals. Increased involvement of geneticists and/or genetic counselors in the care of children with cardiomyopathy would help to standardize the approach to sequence variants and improve care delivery. Focused quality improvement efforts are needed to understand the impact of and potential barriers to the incorporation of genetic expertise in the care of children with cardiomyopathy.

## Supplementary Information

Below is the link to the electronic supplementary material.

Supplementary file1 (PDF 83 kb)
