# Supplementary material for: Genetic Testing Resources and Practice Patterns Among Pediatric Cardiomyopathy Programs
Source: Pediatr Cardiol. 2024 May 7;46(4):798–803. doi: 10.1007/s00246-024-03498-6 (PMC11903623; doi:10.1007/s00246-024-03498-6)
Supplement: Supplementary file 1 — Supplementary file1 (PDF 83 kb) [file 246_2024_3498_MOESM1_ESM.pdf]

Online Resource 1

Article Title: Genetic testing resources and practice patterns among pediatric cardiomyopathy programs
Journal: Pediatric Cardiology
Authors: Justin Godown, Emily Kim, Melanie D. Everitt, Wendy K. Chung, Irene Lytrivi, Sonya Kirmani, Paul Kantor, Stephanie Ware, Jean Ballweg, Ashwin K. Lal, Neha Bansal, Jeffrey Towbin, Steven E. Lipshultz*, Teresa M. Lee.

* **Corresponding author affiliation:**
Steven E. Lipshultz
Professor of Pediatrics
University at Buffalo Jacobs School of Medicine and Biomedical Sciences
Clinical and Translational Research Center
E-mail: slipshul@buffalo.edu

Caption: Data dictionary of the RedCAP survey distributed to providers

Data Dictionary Codebook

Genetics Survey

#	Variable / Field Name	Field Label<br>*Field Note*	Field Attributes (Field Type, Validation, Choices, Calculations, etc.)
Instrument: **Genetics Survey** (genetics_survey)			
1	[record_id]	Record ID	text
2	[center]	What center are you from?	text
3	[experience]	How many years have you been in practice as an attending physician?	dropdown<br>1 < 1 year<br>2 1 to 4 years<br>3 5 to 9 years<br>4 10 to 14 years<br>5 15 to 19 years<br>6 20 or more years
4	[geneticist]	Do you have a geneticist on faculty in the division of cardiology?	yesno<br>1 Yes<br>0 No
5	[genetic_counselor1]	Do you have a genetic counselor within the division of cardiology?	yesno<br>1 Yes<br>0 No
6	[gen_coun]	How do you access genetic counseling? (Check all that apply)	checkbox<br>1 gen_coun___1 Refer to separate genetics team / genetic counselor<br>2 gen_coun___2 Refer to genetic counselor as part of cardiology team<br>3 gen_coun___3 Independently performed by cardiology physician<br>4 gen_coun___4 We do not routinely offer genetic counseling<br>5 gen_coun___5 Other
7	[counseling_txt]<br>Show the field ONLY if:<br>[gen_coun(5)] = '1'	What other methods do you utilize to access genetic counseling?	text
8	[gen_testing]	How do you access genetic testing? (Check all that apply)	checkbox<br>1 gen_testing___1 Refer to separate genetics team<br>2 gen_testing___2 Refer to genetics as part of cardiology team<br>3 gen_testing___3 Independently performed by cardiology physician<br>4 gen_testing___4 We do not routinely offer genetic testing<br>5 gen_testing___5 Other
9	[testing_txt]<br>Show the field ONLY if:<br>[gen_testing(3)] = '1'	What other methods do you utilize to access genetic testing?	text

10	[inhouse]	Do you use in-house genetic testing, commercial genetic testing, or both?	radio<br>1 In house<br>2 Commericial<br>3 Both
11	[wes]	How often do you perform whole exome sequencing on a child with cardiomyopathy?	radio<br>1 Always<br>2 Frequently<br>3 Sometimes<br>4 Not often<br>5 Never
12	[wes_yn]	Are there specific findings that prompt you to consider whole exome sequencing?	yesno<br>1 Yes<br>0 No
13	[wes_txt]<br>Show the field ONLY if:<br>[wes_yn] = '1'	What findings prompt you to pursue testing with whole exome sequencing?	notes
14	[interval]	What is a reasonable interval (in years) to repeat genetic testing in a patient who is phenotype positive but without a known genetic basis?<br>*years*	text
15	[cost]	How often do you find that cost is prohibitive to obtaining genetic testing?	radio<br>1 Almost Always<br>2 Often<br>3 Neutral<br>4 Not often<br>5 Almost never
16	[txt1]	Scenario 1:	descriptive
17	[scenario1_pathogenic]	Genetic testing reveals that your patient who has a clinical diagnosis of cardiomyopathy has a pathogenic variant. Do you offer familial cascade testing for healthy family members for the variant identified?	yesno<br>1 Yes<br>0 No
18	[scenario1_likelypathogenic]	Genetic testing reveals that your patient who has a clinical diagnosis of cardiomyopathy has a likely pathogenic variant. Do you offer familial cascade testing for healthy family members for the variant identified?	yesno<br>1 Yes<br>0 No
19	[scenario1_vus]	Genetic testing reveals that your patient who has a clinical diagnosis of cardiomyopathy has a variant of uncertain significance. Do you offer familial cascade testing for healthy family members for the variant identified?	yesno<br>1 Yes<br>0 No
20	[scenario1_likelybenign]	Genetic testing reveals that your patient who has a clinical diagnosis of cardiomyopathy has a likely benign variant. Do you offer familial cascade testing for healthy family members for the variant identified?	yesno<br>1 Yes<br>0 No
21	[scenario1_benign]	Genetic testing reveals that your patient who has a clinical diagnosis of cardiomyopathy has a benign variant. Do you offer familial cascade testing for healthy family members for the variant identified?	yesno<br>1 Yes<br>0 No
22	[txt2]	Scenario 2:	descriptive
23	[scenario2_path]	You have an asymptomatic patient with no clinical signs of cardiomyopathy who tested negative for a pathogenic variant found in their parent who has cardiomyopathy. Do you discharge them from cardiology clinic?	yesno<br>1 Yes<br>0 No
24	[scenario2_likelypath]	You have an asymptomatic patient with no clinical signs of cardiomyopathy who tested negative for a likely pathogenic variant found in their parent who has cardiomyopathy. Do you discharge them from cardiology clinic?	yesno<br>1 Yes<br>0 No

25	[scenario2_vus]	You have an asymptomatic patient with no clinical signs of cardiomyopathy who tested negative for a variant of unknown significance found in their parent who has cardiomyopathy. Do you discharge them from cardiology clinic?	yesno<br>1 Yes<br>0 No
26	[scenario2_likelybenign]	You have an asymptomatic patient with no clinical signs of cardiomyopathy who tested negative for a likely benign variant found in their parent who has cardiomyopathy. Do you discharge them from cardiology clinic?	yesno<br>1 Yes<br>0 No
27	[scenario2_benign]	You have an asymptomatic patient with no clinical signs of cardiomyopathy who tested negative for a benign variant found in their parent who has cardiomyopathy. Do you discharge them from cardiology clinic?	yesno<br>1 Yes<br>0 No
28	[reliability]	How reliable are genetic testing interpretations (i.e. classification as pathogenic or benign)?	radio<br>1 They are always accurate<br>2 They are mostly accurate, but can rarely change<br>3 They are mostly accurate, but can sometimes change<br>4 They are mostly accurate, but can often change<br>5 They are never accurate
29	[recall_process]	Do you have a process to follow-up with patients who were previously discharged from follow-up if a variant is reclassified that impacts their overall risk?	yesno<br>1 Yes<br>0 No
30	[recall_txt]<br>Show the field ONLY if:<br>[recall_process] = '1'	What mechanism(s) do you use to track patients discharged from cardiology follow-up?	notes
31	[genetics_survey_complete]	Section Header: *Form Status*<br>Complete?	dropdown<br>0 Incomplete<br>1 Unverified<br>2 Complete
